# Relational–ethical transformational leadership in palliative care nursing: A conceptual perspective

**DOI:** 10.1017/S1478951526102363

**Published:** 2026-04-20

**Authors:** Júlia Drummond de Camargo, Ivy de Carvalho Ramalho de Oliveira, João Carlos Geber-Júnior

**Affiliations:** 1 Palliative Care Team, Hospital Samaritano de Sao Paulo, São Paulo, Brazil; 2 Palliative Care Support Team, Hospital Sirio-Libanes, São Paulo, Brazil; 3 Universidade de Sao Paulo Faculdade de Medicina, São Paulo, Brazil; 4 Palliative Care Support Team, Hospital Samaritano Higienópolis, São Paulo, Brazil

## Leadership as a clinical and ethical competency in palliative care nursing

Palliative care unfolds at the intersection of clinical complexity, existential vulnerability, and relational intensity. Within this landscape, nursing is not merely a professional role but a continuous form of clinical presence – one that stabilizes meaning, regulates emotional climates, and translates uncertainty into humane care. Nurses coordinate the everyday choreography of symptom management, family communication, and interdisciplinary alignment, often in circumstances where suffering cannot be eliminated and decisions cannot be reduced to protocols.

Despite this centrality, leadership in palliative care nursing is still frequently framed in managerial terms: staffing models, workflow coordination, and institutional governance. While these dimensions are important, such framing risks obscuring the forms of leadership that most profoundly shape the moral climate of care at the bedside. In the context of serious illness, leadership often emerges through relational influence, ethical discernment, and the capacity to sustain trust within teams navigating uncertainty.

Palliative care is fundamentally team-based. Interdisciplinary collaboration is not an accessory to clinical care; it is the condition under which care becomes coherent. When teams function well, they create a relational container capable of holding uncertainty, processing emotional strain, and enabling shared ethical deliberation. When teams fracture, that fracture is often experienced by patients and families as confusion, abandonment, or escalation of suffering.

A useful entry point into these dynamics is the framework of team dysfunctions described by Patrick Lencioni, in which absence of trust becomes the foundation upon which fear of conflict, lack of commitment, avoidance of accountability, and inattention to results are built (Lencioni 2002). In palliative care, trust rarely emerges from hierarchy or charisma. Instead, it is cultivated through repeated micro-acts: the nurse who names what others hesitate to say, the nurse who asks the clarifying question that prevents a cascade of misunderstandings, the nurse who signals a relational rupture before it becomes a formal complaint.

In this sense, Lencioni’s emphasis on trust as the foundation of team function intersects with the concept of psychological safety described by Amy Edmondson. Psychological safety refers to the shared belief that a team environment permits interpersonal risk-taking without humiliation or punishment (Edmondson 1999). Both frameworks describe relational conditions under which professionals feel able to speak honestly about uncertainty, vulnerability, and ethical concern. In palliative care settings – where decisions unfold under emotional strain and prognostic ambiguity – these relational conditions are not peripheral to clinical work; they are essential to it.

Within this environment, nurses frequently occupy a strategic position in sustaining psychological safety. Their continuous presence at the bedside allows them to detect subtle shifts in relational dynamics long before these tensions become visible in formal clinical discussions. By naming uncertainty, acknowledging vulnerability, and inviting quieter voices into the conversation, nursing leadership helps teams remain capable of open deliberation rather than defensive closure.

Health-care leadership research has consistently associated relational and transformational leadership styles with improved workforce outcomes, including lower burnout and better staff retention (Wong and Cummings 2013; Cummings et al. 2018). Yet palliative care requires a more precise conceptual lens. The central concern is not only organizational performance but also the moral climate within which decisions are made. Leadership in palliative care cannot be adequately understood if relational presence, ethical advocacy, and psychological safety are treated as secondary virtues rather than core clinical competencies.

## Relational presence: The infrastructure of influence

Influence in palliative care is frequently exercised without formal authority. Leadership manifests less through directive instruction and more through relational steadiness – the capacity to remain present without collapsing into urgency, to listen without withdrawing from responsibility, and to regulate the emotional temperature of the clinical encounter.

Communication in contexts of high vulnerability tends to unfold hierarchically: trust precedes emotional engagement, emotional engagement enables shared cognition, and shared cognition supports coherent decision-making. Yet this sequence remains dynamic and iterative rather than strictly linear (Geber-Junior and Forte 2025a). Nurses often become the first to detect when this relational architecture begins to fracture: a patient who withdraws from conversation, a family that agrees without genuine understanding, or a team member who becomes silent in moments requiring reflection.

Relational presence also carries an implicit pedagogical dimension. Teams learn how uncertainty should be handled by observing how leaders respond to it. When uncertainty is treated as incompetence, it becomes hidden. When uncertainty is acknowledged with composure, it becomes discussable. Over time, these interactions shape the epistemic culture of the team – determining whether vulnerability becomes a source of collective insight or a reason for defensive silence.

## Ethical advocacy: Beyond procedural autonomy

Serious illness exposes the limits of purely procedural understandings of autonomy. Pain, delirium, fear, dependency, and family dynamics can destabilize the coherence of individual decision-making. Under these circumstances, ethical practice cannot be reduced to documentation of consent or formal capacity assessments. Instead, it requires interpretive advocacy – an effort to protect dignity by clarifying values, translating clinical realities, and resisting momentum that may inadvertently lead to disproportionate or unwanted interventions.

Recent reflections in palliative care ethics have emphasized that when autonomy fails to provide sufficient moral guidance, clinicians retain obligations grounded in relational responsibility and the prevention of avoidable suffering (Geber-Junior 2025). Nurses frequently function as ethical sentinels within clinical teams. Through their proximity to patients and families, they often perceive when treatment intensity reflects institutional inertia rather than patient priorities.

Empirical evidence suggests that clinicians’ attitudes toward end-of-life decision-making may vary substantially, sometimes revealing discrepancies between professional self-perception and actual decision patterns (de Camargo and Forte 2024). In such contexts, leadership does not consist merely in choosing among options but in sustaining a process of ethical deliberation that remains transparent and relationally grounded.

## Transformational influence and the moral climate of teams

Psychological safety becomes particularly salient in palliative care because the work is emotionally intense and morally complex. Teams must be able to speak openly about uncertainty, disagreement, perceived futility, and the emotional weight of clinical encounters (Edmondson 1999). When such openness is absent, professionals often rely on procedural routines or hierarchical deference to avoid interpersonal risk. The result is not only poor communication but also the emergence of moral distress.

Moral distress is rarely an isolated emotional experience; it is frequently a relational signal that the team’s capacity for ethical reflection has become constrained (Rushton 2018). Leadership in palliative care therefore includes the capacity to transform team dynamics – creating spaces where disagreement can be expressed without rupture and where vulnerability becomes a shared professional resource rather than a liability.

From a medical perspective, the importance of nursing leadership becomes especially visible in moments of prognostic tension. Physicians may carry responsibility for diagnostic and therapeutic decisions, but they often rely on nurses to interpret relational signals within families and within the team itself. In such moments, nursing leadership does not replace medical authority; rather, it stabilizes the deliberative process that makes ethically sound decisions possible.

The ability to remain steady amid prognostic uncertainty – resisting reductionist reliance on prediction while sustaining relational presence – has increasingly been recognized as a defining competency of palliative care practice (Geber-Junior and Forte 2025b). Nurses frequently anchor this steadiness by maintaining continuity across shifts, disciplines, and evolving family dynamics.

## Implications for practice and education

If leadership in palliative care nursing is relational, ethical, and transformational, educational frameworks must address these dimensions explicitly. Competency documents increasingly recognize leadership as a core domain of specialist palliative nursing practice, both in national frameworks (Academia Nacional de Cuidados Paliativos [ANCP] 2022) and in international analyses of palliative care competencies (Hökkä et al. 2021).

Leadership development in palliative care should therefore extend beyond administrative preparation to include relational communication, ethical deliberation under uncertainty, and the cultivation of moral resilience (Reeves et al. 2010; Rushton 2018). Evidence suggests that when nurses are empowered to assume leadership roles, palliative care services become more sustainable and interdisciplinary collaboration improves (Mitrea et al. 2018; Grant and Johnson 2019; Simmons et al. 2020).

At the organizational level, health-care systems that cultivate empathetic institutional cultures and psychologically safe teams are more likely to sustain ethically grounded palliative care while mitigating professional burnout (Cummings et al. 2018; Kerasidou et al. 2021).

## Conclusion

Leadership in palliative care nursing is often exercised quietly – through the steadying of conversations, the protection of dignity, and the mediation of tensions within interdisciplinary teams. When leadership is confined to managerial definitions, these relational and ethical dimensions remain largely invisible despite their central role in shaping patient and family experience.

Viewing leadership through a relational–ethical transformational lens makes visible what experienced clinicians frequently recognize in practice: in environments defined by vulnerability, uncertainty, and emotional intensity, leadership often emerges not through hierarchical authority but through relational steadiness, ethical discernment, and the cultivation of psychological safety.

Recognizing and supporting this form of leadership may strengthen not only the experience of patients and families but also the moral integrity and sustainability of the teams who provide palliative care.

## Competing Interests

The authors declare that they have no competing interests.
